# Identifying New/Emerging Psychoactive Substances at the Time of COVID-19; A Web-Based Approach

**DOI:** 10.3389/fpsyt.2020.632405

**Published:** 2021-02-09

**Authors:** Valeria Catalani, Davide Arillotta, John Martin Corkery, Amira Guirguis, Alessandro Vento, Fabrizio Schifano

**Affiliations:** ^1^Psychopharmacology, Drug Misuse & Novel Psychoactive Substances Research Unit, School of Life & Medical Sciences, University of Hertfordshire, Hatfield, United Kingdom; ^2^Swansea University Medical School, Institute of Life Sciences 2, Swansea University, Swansea, United Kingdom; ^3^Department of Mental Health, ASL Roma 2, Rome, Italy; ^4^Addictions' Observatory (ODDPSS), Rome, Italy; ^5^Department of Psychology, Guglielmo Marconi University, Rome, Italy

**Keywords:** COVID-19, new psychoactive substances, NPS, NPSfinder®, web crawler, drug misuse

## Abstract

COVID-19-related disruptions of people and goods' circulation can affect drug markets, especially for new psychoactive substances (NPSs). Drug shortages could cause a change in available NPS, with the introduction of new, unknown, substances. The aims of the current research were to use a web crawler, NPSfinder®, to identify and categorize emerging NPS discussed on a range of drug enthusiasts/psychonauts' websites/fora at the time of the pandemic; social media for these identified NPS were screened as well. The NPSfinder® was used here to automatically scan 24/7 a list of psychonaut websites and NPS online resources. The NPSs identified in the time frame between January and August 2020 were searched in both the European Monitoring Center for Drugs and Drug Addictions (EMCDDA)/United Nations Office on Drugs and Crime (UNODC) databases and on social media (Facebook, Twitter, Instagram, Pinterest, and YouTube) as well, with a content qualitative analysis having been carried out on reddit.com. Of a total of 229 NPSs being discussed at the time of the pandemic, some 18 NPSs were identified for the first time by the NPSfinder®. These included six cathinones, six opioids, two synthetic cannabinoid receptor agonists (SCRAs), two phenylcyclohexylpiperidine (PCP)-like molecules, and two psychedelics. Of these NPSs, 10 were found to be previously unreported to either the UNODC or the EMCDDA. Of these 18 NPSs, opioids and cathinones were the most discussed on social media/reddit, with the highest number of threads associated. Current findings may support the use of both automated web crawlers and social listening approaches to identify emerging NPSs; the pandemic-related imposed restrictions may somehow influence the demand for specific NPS classes.

## Introduction

The COVID-19 pandemic has been considered as the worst global crisis after the global financial crash of 2007–2008 (1–3). This was caused by massive disruptions in goods' markets and restrictions imposed on individuals' movements (home confinement) followed by the total blocking of air and land travel (January–June 2020) (4). These primary measures generated a substantial economic burden at international, national, and community levels, forcing the general population to face psychological difficulties and behavioral changes (5–8). Of particular concern are people who use drugs (PWUDs) (9, 10). It is well-known how acute or chronic stress can have a pivotal role in the inception of substance abuse and in the worsening of substance use disorders (6, 11).

COVID-19 measures affected the illegal drug markets as well, from production, trafficking, and marketing through to availability and demand. These aspects have been affected in different ways across different countries, with the exception of the retail markets, which have undergone a more homogeneous change. Drug shortages, stockpiling, increase in prices, and reduction in purity were reported across the world (12). This was true especially for the more established drugs like cocaine and heroin, which are produced in specific areas of the world (e.g., South America and Afghanistan) and which rely on open legal commercial routes to be moved around (13). New psychoactive substances (NPSs) (14) encountered a different fate. A diversification of the market was expected between January and June 2020 (12, 15) due to shortages of treatment and classic opiate and opioid drugs (16) pushing users to synthetic available alternatives; lack of precursors for synthetic drugs diverting productions toward new NPS analogs; the economic problems and anxiety caused by the pandemic forcing PWUDs to use cheaper and seek more potent substances; and increased drug e-commerce that followed the restrictions of individual movements (12) facilitating the distribution of NPSs. The expected trend of PWUDs switching to and/or increasingly accessing counterfeit/unknown drugs online represents a serious health threat that should be investigated and monitored.

Monitoring of social media platforms could aid in identifying emerging NPSs during the COVID-19 pandemic. In recent years, social media increased their popularity as interacting platforms, in which users and suppliers of drugs can communicate freely, e.g., about price, purity, pharmacological/toxicological effects, way of administration, dosages of substances, with particular regard to newly introduced/synthesized ones. The analysis of available online information [qualitative analysis (17)] can be an effective tool to understand and identify consumers' needs and decisions and markets supplies and demands' balance. Overall, “social media listening” has been proven to be an effective tool for public health concerns (18).

The aims of the current research were to use a web crawler, NPSfinder®, to identify and categorize emerging NPSs discussed on a range of drug enthusiasts' websites/fora at the time of the COVID-19 pandemic; compare the NPSfinder® results with related listings from the European Monitoring Center for Drugs and Drug Addictions (EMCDDA) and United Nations Office on Drugs and Crime (UNODC) databases (19, 20); screen social media (Facebook, Twitter, Instagram, Pinterest, YouTube) for identified NPSs; and conduct a qualitative analysis (reddit) to better understand the drug market at the time of COVID 19 pandemic.

## Methods

### Identification of Molecules

As better specified in Arillotta et al. (21), NPSfinder® is a crawling/navigating, password-protected, proprietary software, which allows registered researchers only to screen and classify the molecules being identified. Indeed, NPSfinder® automatically scans on a 24/7 basis a range of website addresses/uniform resource locator (URLs) for new/novel/emerging NPSs [see also (22, 23)]. When a novel substance is found, this is added to the growing NPSfinder® database. NPSfinder® was used here to facilitate identification of the range of NPSs discussed online from January to August 2020. Although one could argue that in January and February the European Union and the United States did not have any restrictions in place, the restrictions were at that time clearly in place in China (24), a country that has been suggested as being involved in the production/supplying of both synthetic drugs (NPSs) and synthetic drug precursors (4).

The scanned URLs were representative of online psychonauts' websites/fora and other NPS online resources (see Appendix 1). NPSfinder® was designed by Damicom, an information technology enterprise based in Rome (Italy), to extract a range of information regarding NPSs including chemical and street names, chemical formulas, three-dimensional images, and anecdotally reported clinical/psychoactive effects. The data extracted were automatically stored in an online, restricted-access/password-controlled database. The predominant language was English, but other languages were also considered: Spanish, German, Russian, Italian, Dutch, French, Swedish, and Turkish. From all the data extracted by the web crawler, the range of unique NPSs being identified was assigned to their NPS class, according to the indications taken from a range of literature papers (25–27).

### Comparison Between NPSfinder®, EMCDDA, and UNODC Databases

To assess the possible novelty of NPSfinder® findings, the NPS molecules here identified for the first time by web crawler at the time of the COVID-19 pandemic were compared with entries available from both the EMCDDA's European Database on New Drugs (19) and UNODC Early Warning Advisory on NPS database (20). JMC, a registered user with authorized access to these databases, prepared the listing for the comparison. The comparison was conducted using the International Chemical Identifier Key (InChIKey) (28, 29).

### Social Networks' Analysis

In order to better understand the online overall scenario of those NPSs first identified by the web crawler at the time of the COVID-19 pandemic, a range of social networks (e.g., Facebook; YouTube; Twitter; Instagram; Pinterest, reddit) were investigated as well. An observational qualitative analysis, in the time frame September–October 2020, was here performed, and these social networks were chosen because of their popularity, e.g., number of users. A similar approach has already been used by this research group in other studies (18, 21). A content qualitative analysis was conducted on reddit (30), which is a web-based platform that organize topics into fora known as subreddits, where each discussion is considered a thread. Reddit is well-known for its ability in engaging users and reporting good-quality information on a great variety of topics (30–33); these characteristics make this platform as a very popular source for social listening studies (34–37). Reddit fora entries are anonymous and voluntary. The subreddit called “r/Researchchemical” (38) was initially analyzed for the purpose of this article. “r/Researchchemical” is defined as the subreddit for the discussion of synthetic psychoactive research chemicals, also known as NPSs. When the threads were analyzed, the group had 94,000 members. The terms used for the search were the here newly identified substances, their chemical names, and street names. During the search, other subreddits were deemed relevant to the current study and were hence included in the qualitative analysis, e.g., “r/opiods.RCS,” “r/stims,” “r/noids,” and “r/dissociatives” (39–42). Two independent researchers, with different backgrounds in qualitative research, analyzed independently all the relevant threads. The dataset analysis was conducted manually without the use of any software. The subreddits were screened after the analysis of the data provided by NPSfinder® was concluded and the new molecules identified; to allow optimal collection of qualitative data, no time restrictions were used for the reddit qualitative analysis.

## Results

The NPSfinder® web crawler has been active since November 2017 and to date reported a total of 4,335 NPSs found on the surface web. For this study, data were collected between January and August 2020. During this time frame, the web crawler identified a total of 229 substances (Appendix 2) as being discussed and commented by psychonauts; out of these, and after careful evaluation, 18 were recognized as previously unidentified and new to the NPSfinder®. Proper categorization and descriptive statistics were produced for these 229 molecules (Table 1); most popular NPS categories being commented on included synthetic cannabinoid receptor agonists (SCRAs), synthetic opioids, and cathinones.

**Table 1:** Descriptive statistics of the 229 NPS identified from January to August 2020.

**Total** ***n*** **of NPS identified** **=** **229**	
**NPS class**	**%**
Cannabimimetics	42.80
Opioids	11.35
Cathinones	10.9
Tryptamines	7.86
Gabaergics	7.42
NBOMes	6.55
Phenethylamines	3.93
Hallucinogens	3.06
PCP-like	3.06
PIEDS	1.31
Psychostimulants	0.90
Flys	0.44
Prescribed drugs	0.44

*The molecules identified were divided by NPS class, and the percentage per class calculated*.

The 18 newly identified molecules, categorized in line with both Abdulrahim and Bowden-Jones (25) and Schifano et al. (26), included six cathinones, six opioids, two cannabimimetics, two phenylcyclohexylpiperidine-like substances, one hallucinogen, and one tryptamine. In order to understand if these molecules were not only new but unique to NPSfinder®, a comparison with the UNODC and EMCDDA databases was made. As a result, 10 NPSs were identified as previously unknown/unreported (Table 2). For three of the six new cathinones (Table 2), no information on chemical structure or composition was available, and the molecules appeared here to be totally unknown.

**Table 2:** List of NPS identified for the first time by NPSfinder® from January to August 2020.

**NPS**	**Chemical family**	**Chemical name**	**Description**	**Previously unidentified NPS**	**NPSfinder^®^ identification date**
3M-4F-αPVP	Catinones	1-(4-fluoro-3-methylphenyl)-2-(pyrrolidin-1-yl)pentan-1-one	It is the 3-methyl derivative of 4F-alpha-PVP. Cathinones, which are structurally like 4F-α-PVP, cross the brain-blood barrier effectively (43). No information has been retrieved on its mechanism of action, but it is likely to affect the monoaminergic system, particularly the dopamine transport, as the 4F-PVP. It is a stimulant.	N	23/07/2020
4H-CMC	Cathinones	N.a.	Derivative of 4-CMC s a stimulant drug of the cathinone class.	Y	06/05/2020
MD-PEP/MD-PV8	Cathinones	1-(benzo[d][1,3]dioxol-5-yl)-2-(pyrrolidin-1-yl)heptan-1-one	MDPEP is a stimulant of the Cathinone class, which has been reported as a novel designer drug (44). MD-PEP is the methylendioxy derivative of α-PEP and the higher homolog of α-pyrrolidinohexiophenone (α-PHP), having an extra carbon on the alkyl side chain. No *in vitro* studies are available to assess the activity on the brain but based on previous work the longer alkyl chain may increase its potency (45).	N	06/05/2020
EBK-EBDP	Cathinones	N.a.	EBK-EBDP is probably a mixture of EBK, a new synthetic derivative of βk-EBDP/ephylone, and ephylone itself. On the website where the molecule was first identified by the NPSfinder®, C20H27FN2O3 was the molecular formula reported. The description was then changed to EBK alone. Other chemical formulas are available online for the same compound. It is sold as a potential strong stimulant with powerful psychotic effects.	Y	06/05/2020
HEP	Cathinones	N.a.	HEP belongs to cathinone and amphetamine chemical classes and it is the new HEX-EN replacement.	Y	06/05/2020
A-PCYP	Cathinones	2-cyclohexyl-1-phenyl-2-(pyrrolidin-1-yl)ethanone	A-PCYP is a stimulant drug of the cathinone class that has been sold online as a designer drug. In a series of α-substituted pyrrolidinyl cathinone derivatives developed in 2015, the α-cyclopentyl derivative was found to have around the same potency *in vitro* as an inhibitor of the dopamine transporter as the α-propyl derivative a-PVP, while the α-cyclohexyl derivative α-PCYP was around twice as strong (46).	Y	06/03/2020
4F-MDMB-BICA	Cannabimimetics	Methyl 2-[[1-(4-fluorobutyl)indole-3-carbonyl]amino]-3,3-dimethyl-butanoate	4F-MDMB-BICA is a synthetic cannabinoid structurally similar to 4F-MDMB-BINACA and 5F-MDMB-PICA. 5F-MDMB-PICA is explicitly a Schedule I substance in the United States; 4F-MDMB-BICA is not a scheduled substance (47).	N	23/07/2020
5F-NPB-22	Cannabimimetics	1-(5-fluoropentyl)-8-quinolinyl ester-1H-indazole-3-carboxylic acid	5-F-NPB-22 is an analog of NPB-22 that differs by adding a fluorine atom to the terminal carbon of the alkyl chain (48).	Y	13/06/2020
ETAZENE	Opioids	(2-[(4-ethoxyphenyl)methyl]-N,N-diethyl-1H-benzimidazole-1-ethanamine)	Etazene was notified as an NPS on 1 June 2020 by Poland (49). The substance belongs to the 2-benzylbenzimidazole group of synthetic opioid analgesics; It is less potent than isonitazene but still almost 70 time more potent than morphine (50, 51).	N	23/07/2020
METODESNITAZENE	Opioids	N,N-diethyl-2-(2-(4-methoxybenzyl)-1H-benzo[d]imidazol-1-yl)ethan-1-amine;	Metodesnitazene is a 2-benzylbenzimidazole. It is structurally related to etonitazene (Schedule I of the 1961 United Nations Single Convention on Narcotic Drugs), with the presence of an ethoxy group instead of the methoxy and the absence of the nitro group at the 5 position. The analgesic activity of the 2-benzylbenzimidazole appears to be related to the substitution at the benzyl moiety with para substitution showing higher activity (52).	N	23/07/2020
FLUNITAZENE	Opioids	N,N-diethyl-2-[(4-fluorophenyl)methyl]-5-nitro-1H-benzimidazole-1-ethanamine	It is a novel opioid of the 5-nitro-2-benzylbenzimidazole family that shares the same structure as Clonitazene but with a fluorine atom instead of the chlorine in para to the phenyl ring.	Y	23/05/2020
BRORPHINE	Opioids	1-[1-[1-(4-bromophenyl)ethyl]-4-piperidinyl]-1,3-dihydro-2H-benzimidazol-2-one	Brorphine is a piperidine benzimidazolone (3-piperidin-4-yl-1*H*-benzimidazol-2-one). It shares structural similarities with the internationally controlled narcotic analgesic bezitramide and with the benzimidazole opioids isotonitazene and etazene. However, the latter cannot be considered close derivatives (49).	N	18/03/2020
DIPHENPIPENOL	Opioids	3-[2-[4-(2-methoxyphenyl) piperazin-1-yl]-2-phenylethyl]phenol	Diphenpipenol was invented in the 1970s by Dainippon Pharmaceutical Co (53). It is an opioid analgesic, derivative of 1-substituted-4-(1,2-diphenylethyl)piperazines. It is related to MT-45 and AD-1211, being the most potent compound in the series. The (S) isomer has 105 times the potency of morphine in animal studies (54). This makes it a similar strength to fentanyl and consequently diphenpipenol can be considered a threat to life expected to cause respiratory depression, sedation, itching, nausea and vomiting upon consumption.	Y	20/08/2020
NORTILIDINE	Opioids	ethyl-2-(methylamino)-1-phenylcyclohex-3-ene-1-carboxylate	Nortilidine is the major demethylated active metabolite of tilidine. The racemate has opioid analgesic effects roughly equivalent in potency to that of morphine (55). The drug also acts as a dopamine reuptake inhibitor (26).	N	20/08/2020
3-CL-PCP	PCP-like	1-[1-(3-Chlorophenyl )cyclohexyl]piperidine	3-Chlorophencyclidine (3-CL-PCP) is a dissociative anesthetic drug with hallucinogenic and sedative effects that has been sold as a research chemical. It has comparable potency to phencyclidine but slightly different effects. This is due to its altered binding profile at various targets, particularly being somewhat more potent as an NMDA antagonist while having around the same potency as a dopamine reuptake inhibitor.	Y	23/07/2020
3-F-PCP	PCP-like	1-[1-(3-Fluorophenyl) cyclohexyl]piperidine	3-F-PCP is a dissociative hallucinogen of the aryl cyclohexylamine class related to phencyclidine (PCP) which has been sold online as a designer drug. It is the fluorinated analog of the 3-MeO-PCP, substance listed in UK as Class B of the Misuse of Drugs Act (1971). No *in vitro* studies have been found for this compound but due to the similarity with 3-MeO-PCP it should act acts mainly as an NMDA receptor antagonist interacting with the sigma σ1 receptor and the serotonin transporter as well.	Y	23/07/2020
1F-LSD	Hallucinogens	(6aR,9R)-9-(diethylcarbamoyl)-7-methyl-6a,7,8,9-tetrahydroindolo[4,3-fg]quinoline-4(6H)-carboxylic acid	1-formyl-lysergic acid diethylamide is a chemical analog of ALD-52, which is a formyl group on position 1 instead of an acetyl. No information on potency is available.	Y	23/07/2020
5-CHLORO-DMT	Tryptamines	2-(5-Chloro-1H-indol-3-yl)-N,N-dimethylethan-1-amine	5-chloro-N,N-dimethyltryptamine is a novel, naturally occurring tryptamine found in certain species of deep marine sea sponges, including Smenospongia aurea and Smenospongia echina. It is closely related to 5-bromo-DMT. It was assayed for the *in vitro* serotonin binding receptors. It showed high nanomolar affinity to several serotonin receptors subtype. The highest affinity was observed	N	03/08/2020

All the 18 molecules identified were identified across a variety of vendor sites (56–59). Only few hits were obtained by the analysis of Facebook, YouTube, Instagram, and Pinterest, whereas on Twitter 7 of these 18 molecules were identified and commented on. Of these, four were identified on the seller's profiles only (MFPVP, MD-PV8, 5F-NPB-22, and nortilidine): two in posts/discussions (A-PCYP, 4F-MDMB-BICA), and only one was mentioned within a trip report (1F-LSD). Three molecules (5F-NBP-22, MFPVP, Etazene) were found on Facebook, Pinterest, and Instagram on the sellers' profiles, and only one (3-Cl-PCP) was found on YouTube (e.g., within a trip report).

Conversely, the outcome of the qualitative analysis conducted on the subreddits for these 18 substances provided here more comprehensive results. Across all subreddits, threads were found for all but two of the 18 molecules (i.e., HEP and 5F-NPB22). The subreddit called “r/Researchchemical” included most threads for all the NPS classes, although opioids seemed to be discussed more often on their dedicated subreddit (“r/opiods.RCS”). Overall, the threads/posts relating to these NPSs were entered by redditors starting in a period that range from 2018 to beginning of 2020; whenever possible, the first data post was here identified and analyzed. Overall, older threads were found to be less informative (e.g., in terms of effects, toxicity, dosage, and ways of administrations) than most recent ones. The threads focusing on trips, effects, and routes of administration seemed to attract the most interest, whereas most popular NPSs included opioids and cathinones, followed by PCP-like molecules and psychedelics.

The total number of threads focusing on opioids was 188, of which 84 were on brorphine and 85 on etazene. The oldest thread related to diphenpipenol and was dated August 2019, whereas most recent threads focused on both brorphine and etazene. Etazene presented with the highest number of posts associated with a thread, followed by brorphine and fluonitazene. Among the opioid threads, the highest number of posts was identified as those discussing/comparing several synthetic opioids, with particular attention to tolerance and dosages (Table 3).

**Table 3:** Most popular reddit threads for each of the NPS classes identified by the NPSfinder® between January and August 2020.

**NPS class**	**Total threads**	**Most discussed threads**	**No. of posts**
Opioids	118	“Etazene Taper from Methadone Q's”	70
		“Etazene extinct?”	55
		“Brorphine and Metodesnitazene.”	47
		“Fluonitazene”	35
		“A few Interesting Opioid Molecules I came up with and the respective Swiss Target Prediction”	63
		“which rc opioid?”	38
Cathinones	101	“Raning about A-PCyP”	68
		“a-PCyP: just say no to snorting it”	33
		“MFPVP/mf-pvp/3m-4f-pvp REPORT”	30
		“MDPEP (as known as MD-PV8) turns out do be a good replacement cathinone in terms of duration & effects similar to MDPV or MDPHP”	22
PCP-like	21	“3-F-PCP and World Domination—Phase Two Underway”	91
		“New Stuff's comin'-−3-F-PCP,”	81
		“3-chloro-pcp! it's really nice!”	50
		“My Initial Impressions of 3-CL-PCP”	32
Psychedelics	10	“1F-LSD 100 mcg (A New Lysergamide)—First Trip Report”	129
		“1F-LSD (150 μg sublingual): Novel lysergamide report”	80
Synthetic cannabinoids	5	“5-Bromo-DMT and 5-Chloro-DMT coming soon I think”	34
		“Warning 4F-MDMB-BICA caused 11 deaths in Hungary”	120
		“4f mdmb bica super potent”	16

*The threads can be found in the following subreddits: “r/Researchchemical,” “r/opiods.RCS,” “r/stims,” “r/noids,” and “r/dissociatives”*.

The total number of threads identified for cathinones was 101, of which 70 threads were for A-PCYP only. The oldest thread was dated January 2019 for EBK-EBDP, whereas the most recent focused on MFPVP. Most posts were associated with A-PCYP, commenting on trip reports and effects and routes of administration (Table 3). For the two PCP-like molecules, a total of 21 threads were identified with discussions that started in March 2020. The highest number of posts related to 3-F-PCP (Table 3). Some 10 threads were associated with the psychedelics 1F-LSD and 5-Cl-DMT; related discussions started in January 2019 for 1F-LSD (Table 3). Finally, only five threads were here associated with SCRAs; related discussions started in August 2020, and the latest one in October 2020.

A selection of anecdotal data from the related subreddits referring to the 18 NPSs' availability, desired effects, side effects, routes of administration, onset of action, etc., is reported in Table 4.

**Table 4:** Information gathered from the qualitative analysis of reddit.com for the 18 molecules identified by NPSfinder®.

**NPS**	**Description**	**Reddit threads time frame**
**Opioids**		
Brorphine	Novel opioid/research chemical opioid of psychonauts' interest. It is actively discussed on forums with comparisons and trip reports	May 2019–October 2020
	“Looks like a bastard child of the active metabolite of bezitramide, and benzylfentanyl. Bezitramide was pulled off the market due to overdoses. This one makes me nervous, too close to fentanyl to be safe”	
	“It appears the therapeutic index is quite low, from anecdotal reports. Which means that the overdose level is not very much higher than the level needed to get high. I've also been told the high isn't great and is not very euphoric compared to many other opiates. I would stay far away from this stuff, and if you must use RC opioids, stick with the tried and true and relatively safe O-desmethyltramadol.”	
Diphenpipenol	Novel opioid discussed by psychonaut with a potency and efficacy that are relatively low respect other novel synthetic opioids	August 2019–June 2020
	“It was total waste of money, completely inactive”	
	“Diphenpipenol review (…) It seems to be basically inactive. I have tried nasal vaping and iv use to no avail. Vaping doesn't work at all, burns product and smoke is extremely harsh on lungs. Burns a lot intranasally as well. Disappointed.”	
Etazene	Recently sold as designer drug and identified in June 2020. It is classified as novel opioid/research chemical opioid. It is a substance of interest to psychonauts.	November 2019–October 2020
	“it is a very strong opioid. It differs slightly in action from classic opioids. It is a molecular speedball. If you take little, you feel everything at once: euphoria, speed and relaxation. If you take more, euphoria is growing, but the opiate action profile is getting stronger, until you finally fall into opioid drowsiness. Compared to isotonitazene and fentanyl, it was hardly seen to cause respiratory depression.”	
	“If you don't have an opioid tolerance and aren't an experienced opioid user, this is not one you would want to purchase. The reason it is dosed in nasal sprays is for volumetric dosing: basically you can be sure that each press of the nasal spray is a certain amount, and it can be in micrograms. The difference between fine and overdosed/dead is under 10 mg, which you can't even eyeball.”	
Fluonitazene	Very scares information	May–September 2020
	“Some Chinese vendor spat out a new nitazene. Fluonitazene should be stronger than clonitazene, about 20–40x morphine. No idea if it's legit” (39).	
	“I went on a 23 h binge—the legs on this thing is pretty good, but it's also pretty sedating and not particularly euphoric. All in all, I had a good time, but I ran out just before the 24 h mark. I felt really sad afterwards, and depression-slept for 10 h straight, but after waking up this morning, I was glad that I threw most it out”	
Metodesnitazene	Recently sold as designer drug. It is classified as novel opioid/research chemical opioid. Even though there are a few trip reports, it is a psychonaut substance of interest	January–May 2020
	“meto-des-nitazene: Has a dosage like morphine. There are more interesting substances in this group. I do not recommend buying” (42)	
	“It wasn't until today, taking a 200 mg!! ……. that I felt anything………a minor codeine-like high right now. It started with minor warmth in the head, not the typical opioid warmth we know and love but like I had been out in the sun for 10 min. From there it only progressed a little bit, giving me a very minor and not strong high. In conclusion, this drug is absolutely not worth buying or looking into.”	
Nortilidine	Recently sold as a designer drug, it is a tilidine active metabolite with potential attractive effects for psychonauts.	April 2018–July 2020
	“It's actually as strong as Morphine so it would be a worthwhile RC (…) I thought tilidine was a German speaking countries only thing. It's also marketed in Belgium Bulgaria and South Africa.”	
**Cathinones**		
3M-4F-αPVP	Better known and mostly discussed on forums as “MFPVP” and widely traceable on the surface web (forums and sellers).	May-September 2020
	“R new flakka replacement….PURE PARANOIA on the comedown, its just awful without benzos, with them, its similar to a good amphetamine experience or Molly-like experience”	
	“MFPVP/4F-3M-PVP is worth a try It's actually surprisingly good, though pretty mild” (40)	
	“I noticed it is the only chem that vaping as an r.o.a doesn't just trigger a weird head pressure and make me annoyed! I didn't try oral, never really do for anything, although not that I say that I am wondering why! It is a very short lasting rush, even when IV'd (which is not really a good idea, especially if it'd new, but probably in general, but.self-destruction is human nature I guess. I find insuffliation to be the best happy medium, vaping does seem to add side effects to almost every chem (Hexen especially), but is likely specific to the individual as most effects appear dependent upon. Njot quite sure of dosage”	
4H-CMC	Unknown compound	June 2019
	“Any experience with/knowledge of 4H-CMC?. the only information they're giving is that ‘it's not 4-CMC.' Request for an IUPAC was not met.”	
A-PCYP	A very well-known, discussed and apparently appreciated cathinone. Info are available on various websites (e.g., Isomer Design, Wikipedia), on social networks and vendor websites	November 2019–September 2020
EBK-EBDP	Unknown compound	June 2019
	“A blend of two relatively novel compounds needs purification. EBK (BK-EBDP analo) + Eutylone blend. Any ideas how to do this?”	
HEP	No information on the web retrieved.	N.a.
MD-PEP/MD-PV8	Substituted cathinone traceable in some surface web vendor website. Apparently mostly unknown to the psychonauts. According to some users, probably it is “MD-PV8” or a “MD-PHP analog” and it is shipped from China.	May 2019–April 2020
	“MDPEP waste. MD PV8 cytotoxic almost 0 euphoria don't try!)”	
	“It's less potent, less euphoric and more dangerous than most other pyros. There was a guy that died from consuming 800 mg in one night”	
**Cannabimimetics**		
4F-MDMB-BICA	Described on some vendor websites. Sold as synthetic cannabinoid. User feedback not available on surface web.	August–October 2020
5F-NPB-22	Described on some vendor websites. Sold as synthetic cannabinoid. User feedback not available on surface web.	N.a.
**PCP-like**		
3-CL-PCP	Psychonauts seems to be interested in it. Few trip reports, being a new chemical. Widely traceable on the surface web on vendor websites and some social media.	July–October 2020
	“3-chloro-pcp! it's really nice! So I tested 3-cl-pcp and it is similar to 3-meo-pcp with the hypomania and stimulation, but also has a really comfy calm euphoria similar I believe if Im remembering correctly to ketamine. Lasts like 6 h with stimulation for longer. but the stimulation is nice this time imo. 25 mg is a very mild dose. 60 mg was really fun! IT IS WAAAAY BETTER THAN 3-FLUORO-PCP!”	
3-F-PCP	Various trip reports so far. PCP related substance, it has intrigued psychonauts since its introduction on the market.	March–October 2020
	“3 fluoro pcp is amazing. Got a sample of this last week with another order from china. And wow (…) It is one of the cleanest, clearest, smoothest euphoria from any disso I have tried (and I've tried em all basically) Its stimulating yet relaxed. Chill yet energizing. Not confusing in the least. Highly recommend trying this chemical whenever you can get your hands on it. ROA was IV in dosages of 5–15 mg”	
	“30–40 mg orally is where it becomes enjoyable (…) Ummm like if they wanted to make a tame version of pcp for hospital anesthesia without totally tripping people out at the same time (…) Lasts about 2–3 h, but leaves you with a really long lasting stimulation which can be either bad or good depending on the situation.”	
**Psychedelics**		
1F-LSD	Little data so far. It seems to be a psychonauts' substance of interests, especially for those who like to enjoy psychedelic trips	January 2019–October 2020
	“It had a distinct visual character—while for me many other lysergamides create aforementioned discrete and distinct contained visuals following animal forms and resembling various kinds of indigenous American art, 1F-LSD had a character of more organic, free flowing visuals, with less color. The headspace was subtle and euphoric, given to earnest but pleasant and merciful introspection, with a lot of holistic reflection on contentedness and the passage of time.”	
5-CHLORO-DMT	Not relevant info yet.	February–October 2020
	“There are a couple of vendors which sell these two tryptamines now, but there hasn‘t been any trip report about 5-Chloro-DMT released yet and there are only two reports about 5-Bromo-DMT”	

*The subreddits analyzed were “r/Researchchemical,” “r/opiods.RCS,” “r/stims,” “r/noids,” and “r/dissociatives”. (Note: Current anecdotal data refer to a range of redditors' entries which may be contribute to illustrate the level of the debate relating to the index NPS; no editing has been carried out)*.

## Discussion

The present article provided a unique insight into the world of the NPSs being discussed online at the time of the COVID-19 pandemic. The results presented here for the activity of the NPSfinder® web crawler showed the importance of the web as an essential source to understand and assess the NPS phenomenon (60). Indeed, previous research from our group (21–23, 61) showed how the overall numbers of synthetic cathinones, opioids, benzodiazepines, and SCRAs identified online since the launch of NPSfinder® (November 2017) were higher than those reported to, and listed by, both the EMCDDA and the UNODC. Some 10/18 of the molecules here identified and commented online at the time of the pandemic were unknown/unreported NPSs (19), and this may highlight the potential of automated web crawlers to accurately describe the evolving drug scenarios.

The 18 molecules identified were distributed across the different NPS classes, roughly in line with international data (4, 62, 63). Conversely, in contrast with recent annual reports indicating an increase in designer/ “exotic” benzodiazepines' number, type, and availability (64, 65), these molecules did not feature here between those first identified by NPSfinder® at the time of the COVID-19 pandemic. One could, however, argue that with the COVID-related disruption of medical/health services (66–68), patients, as it has happened in the United Kingdom, may well have managed to get access to large prescription batches of prescription drugs, hence the decreased need to access the web for designer alternatives. Indeed, an increase in the consumption of prescription benzodiazepines has recently been reported (69). Of the 229 NPSs being discussed online at the time of the pandemic, however, synthetic opioids were featured just after SCRAs and were here one-third (e.g., 6/18) of those first identified by the web crawler at the time of the pandemic (49, 65).

While the data obtained from Twitter, YouTube, Facebook, and other social media were few and could not be used here as a solid base for data interpretation, the parallel qualitative analysis conducted on subreddits seemed to have well-supported the web crawler findings. A massive interest toward synthetic opioids was confirmed by the analysis of reddit entries, and this may have paralleled the shortage of heroin (4, 16, 70–72).

The development of new synthetic opioids could worsen the already worrisome worldwide opioid crisis (73–75). NPS opioids are very powerful analgesics, characterized by severe adverse effects such as abuse liability and respiratory depression (21). Although none of the opioids first identified by NPSfinder® at the time of the pandemic was a structural analog of fentanyl (76, 77), all potentially present with a similar threat to public health (63) and are reported to be far more potent than morphine (50, 54, 55). New synthetic opioids were derivatives of different chemical families, such as 2-benzylbenzimidazole and 1-substituted-4-(1,2-diphenylethyl)piperazines. Diphenpipenol, for example, presents with a similar strength to fentanyl, although was anecdotally reported here as “inactive” and “a total waste of money.” It is possible that, although advertised as diphenpipenol, the actual compound made available for purchase was one of its structural isomers with a much weaker opioid activity (78). The recent emergence of this group of opioids may suggest a step back from fentanyl, arguably as a result of control measures introduced in the United States and China in 2019 (51).

The synthetic cathinones' group was followed here in terms of popularity on reddit. Differently from the synthetic opioids, this result is slightly unexpected. In line with the increase reported in the number of newly identified cathinones for 2019 (63), three of the six cathinones identified as first discussed at the time of the pandemic were previously unknown. Furthermore, we recorded here an intense increased vendors' activity to possibly counteract, with cathinones, the threatened/expected shortage of cocaine (13). However, the possible presence on the market of these new compounds is a reason of concern, because of their well-known severe side effects (e.g., paranoia, cognitive impairment, hallucinations, violence, and suicidal thoughts) (79, 80) that could worsen existing depression and trigger low mood induced by COVID-19 (6).

Psychedelic and PCP-like molecules, despite being lower in number compared to the other chemical classes identified, were also discussed at the time of the pandemic. One could argue that these categories of drugs, indeed very popular within the psychonauts' niche scenario (81, 82), were self-administered in a private context, helping to evade the stress, discomfort, and uncertainties associated with COVID-19.

### Limitations

It must be emphasized here that the NPSfinder® crawling activity and the further manual analysis was conducted here only on the surface web. Further studies from our group will focus on the deep web and darknet, as there may be more information in the hidden web (83). Moreover, the present NPSfinder® findings related mostly to psychonaut and vendor websites and may not represent the entirety of those NPSs debated/discussed/mentioned online. Furthermore, one could argue that of the 18 new NPSs identified here, only 10 were not in EMCDDA and UNODC databases at the time of the analysis, and hence only 10 were new. Conversely, as in previous articles (21–23), we thought that it was useful to provide the reader with comparison of current with existing data at the time of the analysis provided by reliable NPS databases such as the EMCDDA and UNODC. Although eight NPSs were already identified by these databases, they were discussed online by the psychonauts at the time of the pandemic, and hence they were grouped together with the “new” ones. Of course, because of a range of methodological differences, it may happen that not all the substances reported by the UNODC and the EMCDDA are identified by the NPSfinder, and *vice versa* (22). However, the evaluation of the NPSfinder performances was beyond the scope of the current article.

Regarding the qualitative analysis, one could argue that people posting on the subreddits may not be representative of the wide community of PWUDs or high-risk groups (e.g., homeless, individuals from deprived areas, adolescents/youth, etc.). Another limitation related here to the sole use of English as the language chosen for the reddit analysis; this may have been associated with levels of loss in data collection. Languages such as Chinese, Japanese, and Arabic were not here included in the NPSfinder® searches, but this will occur in future works. Qualitative methods are at times generally questioned for reliability and objectivity. Finally, the analysis of data originating from the subreddits was conducted manually without the use of any *ad hoc* software, and this may have introduced levels of bias. To overcome this issue, two professionals separately analyzed here the data.

## Conclusions

The analysis of the web presented here has a potential to identify a range of new and previously unidentified/unreported NPSs, with the chance of providing information on current drug trends. The ability of monitoring the net had been proven useful in detecting possible changes in the online drug markets that can reflect the real-world situation during such unprecedented times.

The 18 new NPSs identified in this study, and the related threads analyzed here, showed an appetite for synthetic drugs during a period of negative economic trend imposed by the COVID-19 pandemic. Results from the qualitative analysis on reddit confirmed how opioids represented the most discussed class of NPSs and the one that should keep getting more attention from international health and regulatory bodies. We noticed that while some of these opioids made their first appearance in redditors' discussions before COVID-19, related posts and experiences increased during the first semester of 2020. One could argue that present findings may be consistent with the observation that, in times of stress and crisis, PWUDs prefer drugs that can be used/experienced in solitude to escape the anxiety, boredom, uncertainty, and discomfort generated by the COVID-19 pandemic (84). Uncertainty and fear caused by this unprecedented crisis could push vulnerable people toward dangerous/risky behavior and increased drug consumption. Hence, entry into the drug markets of new and perhaps very potent NPSs is a clear reason of concern.

It is of interest that some of the emerging NPS molecules here described received the attention of redditors even a few months before the start of the pandemic; hence, further studies should combine the use of both web crawlers and social listening data to optimally identify drug scenarios' modifications. These studies, based on a thorough qualitative analysis of both psychonauts' fora and social media, should better assess not only the molecules mentioned by NPS enthusiasts, but also the users' understanding of the pharmacological characteristics of these same molecules.

Finally, the current findings indeed support and highlight the potential and added value of automated web crawlers such as the NPSfinder® in scanning the web and retrieve data in an easy and time-effective way. At present, when a second wave of COVID-19 is generating further lockdown measures, it will remain to be seen if online drug sales and/or increased popularity of some NPSs will persist and influence future drug consumption patterns (63).

## Data Availability Statement

The original contributions presented in the study are included in the article/Supplementary Material, further inquiries can be directed to the corresponding author.

## Author Contributions

VC and FS conceived the idea of the manuscript and coordinated the whole project. VC and DA carried out the process of both data collection and systematization. VC performed the literature searching, the analysis of data, and drafted the manuscript. JC provided data from the EMCDDA and UNODC databases for the purposes of this research. AV supervised the word done with NPSfinder®. FS, AG, and JC contributed to the literature overview and the drafting of the paper. All authors contributed to the articles and approved the submitted version.

## Conflict of Interest

The authors declare that the NPSfinder^®^ web-crawler was provided by Damicon srl. The remaining authors declare that the research was conducted in the absence of any commercial or financial relationships that could be construed as a potential conflict of interest.
