# Supplementary material for: Identifying New/Emerging Psychoactive Substances at the Time of COVID-19; A Web-Based Approach
Source: Front Psychiatry. 2021 Feb 9;11:632405. doi: 10.3389/fpsyt.2020.632405 (PMC7900492; doi:10.3389/fpsyt.2020.632405)
Supplement: Supplementary file 1 [file Data_Sheet_1.docx]

Appendix 1: List of websites monitored by the NPSfinder^®^ web crawler, January-August 2020, surface web only

| N | Website name |
| --- | --- |
| 1 | Avalonmagicplants.com |
| 2 | Azarius.net |
| 3 | Bluelight.org |
| 4 | Bluemorphotours.com |
| 5 | Cannabis.net |
| 6 | Chemeurope.com |
| 7 | Committedpsychonaut.tumblr.com |
| 8 | Consolidated Index of Controlled Substances |
| 9 | Daath.hu/psychonauts |
| 10 | Dancesafe.org |
| 11 | Deviantart.com/psychonaut-a |
| 12 | Druglibrary.org |
| 13 | Drugs.tripsit.me |
| 14 | Drugs-forum.com |
| 15 | Drugs-plaza.com |
| 16 | Dutch-headshop.eu |
| 17 | Ecstasydata.org |
| 18 | Elephantos.com |
| 19 | Energycontrol.org |
| 20 | Entheogen-network.com/forums |
| 21 | Erowid.org |
| 22 | Eusynth.org |
| 23 | Everything2.com/title/Psychonaut |
| 24 | Fungifun.org |
| 25 | Hedweb.com |
| 26 | Hipforums.com/forum |
| 27 | Isomerdesign.com |
| 28 | Knehnav.home.xs4all.nl |
| 29 | Kratomshop.com |
| 30 | Legal-high-inhaltsstoffe.de |
| 31 | Mindstates.org |
| 32 | Mycotopia.net |
| 33 | Natmedtalk.com |
| 34 | Npsproject.eu |
| 35 | Peyote.com/peyolink.html |
| 36 | Psychedelic-library.org |
| 37 | Psychonaut.ca |
| 38 | Psychonaut.fr |
| 39 | Psychonautdocs.com |
| 40 | Psychonautwiki.org |
| 41 | Psyconauts.tripod.com |
| 42 | Reddit.com and drug-related subreddits (e.g. Reddit.com/r/Psychonaut/; Reddit.com/r/shroomers/) |
| 43 | Shayanashop.com |
| 44 | Sjamaan.com |
| 45 | Tripzine.com |
| 46 | Tryptamind.com |
| 47 | Urban75.net |
| 48 | Wikipedia List of designer drugs |
| 49 | Zamnesia.com |
