# Supplementary material for: Identifying New/Emerging Psychoactive Substances at the Time of COVID-19; A Web-Based Approach
Source: Front Psychiatry. 2021 Feb 9;11:632405. doi: 10.3389/fpsyt.2020.632405 (PMC7900492; doi:10.3389/fpsyt.2020.632405)
Supplement: Supplementary file 2 [file Data_Sheet_2.docx]

Appendix 2: List of the 229 substances identified by NPSfinder^®^ from January to August 2020. Some compounds were identified multiple times because discussed on multiple websites.

| NPS class | Molecule denomination | NPS class | Molecule denomination |
| --- | --- | --- | --- |
| Cannabimimetics | FUB-AMB | Cathinones | MD-PEP |
| Cannabimimetics | SDB-005 | Cathinones | EBK-EBDP |
| Cannabimimetics | SGT-263 | Cathinones | HEP |
| Cannabimimetics | 5F-ADB | Cathinones | A-PCYP |
| Cannabimimetics | 5F-AKB57 | Cathinones | MFPVP |
| Cannabimimetics | MAB-CHMINACA | Cathinones | N-ETHYL-(NOR)-PENTEDRONE |
| Cannabimimetics | MMB-CHMINACA | cathinones | 3-MMC |
| Cannabimimetics | NM-2201 | Fly | 2C-B-FLY 10MG PELLETS |
| Cannabimimetics | SGT-151 | Gabaergics | BROMAZOLAM 2.5MG PELLETS |
| Cannabimimetics | 5CL-ADB-A | Gabaergics | CLONAZOLAM 0.5MG PELLETS |
| Cannabimimetics | EG-2201 | Gabaergics | CLONAZOLAM POWDER |
| Cannabimimetics | EG-018 | Gabaergics | DICLAZEPAM POWDER |
| Cannabimimetics | EG-2201 | Gabaergics | ETIZOLAM 1MG BLOTTERS |
| Cannabimimetics | NNL-1 | Gabaergics | ETIZOLAM 1MG PELLETS |
| Cannabimimetics | 4F-MDMB-BINACA | Gabaergics | ETIZOLAM POWDER |
| Cannabimimetics | 5F-MN-18 | Gabaergics | FLUALPRAZOLAM 1MG PELLETS |
| Cannabimimetics | 5F-NPB-22 | Gabaergics | FLUALPRAZOLAM POWDER |
| Cannabimimetics | 5F-SDB-005 | Gabaergics | FLUNITRAZOLAM 0.25MG BLOTTERS |
| Cannabimimetics | ADB-BINACA | Gabaergics | FLUNITRAZOLAM 0.25MG PELLETS |
| Cannabimimetics | AMB | Gabaergics | FLUBROMAZOLAM |
| Cannabimimetics | BIPICANA | Gabaergics | FLUCLOTIZOLAM 0.5MG BLOTTERS |
| Cannabimimetics | FUB-NPB-22 | Gabaergics | FLUNITRAZOLAM POWDER |
| Cannabimimetics | NPB-22 | Gabaergics | NORFLURAZEPAM POWDER |
| Cannabimimetics | 5F-AB-PICA | Gabaergics | PYRAZOLAM |
| Cannabimimetics | 5F-PY-PICA | Gabaergics | ETIZOLAM |
| Cannabimimetics | AB-BICA | hallucinogenic | 2-FDK |
| Cannabimimetics | ADB-BICA | Hallucinogens | 1F-LSD |
| Cannabimimetics | NNL-2 | hallucinogens | PSILOCYBIN & PSILOCIN |
| Cannabimimetics | JWH-320 | hallucinogens | 1B-LSD |
| Cannabimimetics | FUB-NPB-22 | hallucinogens | 1CP-LSD |
| Cannabimimetics | NPB-22 | hallucinogens | 1P-LSD 100MCG |
| Cannabimimetics | 5F-AB-PICA | hallucinogens | AL-LAD 150MCG BLOTTERS |
| Cannabimimetics | 5F-PY-PICA | NBOMe | 25B-NBF |
| Cannabimimetics | AB-BICA | NBOMe | 25C-NBF |
| Cannabimimetics | ADB-BICA | NBOMe | 25IP-NBOME |
| Cannabimimetics | NNL-2 | NBOMe | 25H-NBOME |
| Cannabimimetics | JWH-320 | NBOMe | MESCALINE-NBOME |
| Cannabimimetics | EG-018 | NBOMe | 25B-NBF |
| Cannabimimetics | EG-2201 | NBOMe | 25C-NBF |
| Cannabimimetics | NNL-1 | NBOMe | 25IP-NBOME |
| Cannabimimetics | 4F-MDMB-BINACA | NBOME | 25H-NBOME |
| Cannabimimetics | 5F-MN-18 | NBOMe | 25B-NBF |
| Cannabimimetics | 5F-NPB-22 | NBOMe | 25C-NBF |
| Cannabimimetics | 5F-SDB-005 | NBOMe | 25IP-NBOME |
| Cannabimimetics | ADB-BINACA | NBOMe | 25H-NBOME |
| Cannabimimetics | AMB | NBOMe | MESCALINE-NBOME |
| Cannabimimetics | BIPICANA | Opioids | ISOTONITAZENE |
| Cannabimimetics | EG-2201 | NBOME | MESCALINE-NBOME |
| Cannabimimetics | NNL-1 | opiods | DIPHENPIPENOL |
| Cannabimimetics | 4F-ADB | opiods | NORTILIDINE |
| Cannabimimetics | 5F-MN-18 | Opioids | P-HYDROXY-BUTYRYLFENTANYL |
| Cannabimimetics | 5F-NPB-22 | Opioids | BRORPHINE |
| Cannabimimetics | 5F-SDB-005 | Opioids | U-49900 |
| Cannabimimetics | ADB-BINACA | Opioids | U-51754 |
| Cannabimimetics | AMB | Opioids | BRORPHINE |
| Cannabimimetics | BIPICANA | Opioids | U-49900 |
| Cannabimimetics | FUB-NPB-22 | Opioids | U-51754 |
| Cannabimimetics | 5F-AB-PICA | Opioids | 2-METHYL-AP-237 |
| Cannabimimetics | 5F-PY-PICA | Opioids | P-HYDROXY-BUTYRYLFENTANYL |
| Cannabimimetics | AB-BICA | Opioids | BRORPHINE |
| Cannabimimetics | ADB-BICA | Opioids | U-49900 |
| Cannabimimetics | NNL-2 | Opioids | U-51754 |
| Cannabimimetics | JWH-320 | Opioids | P-HYDROXY-BUTYRYLFENTANYL |
| Cannabimimetics | EG-2201 | Opioids | BRORPHINE |
| Cannabimimetics | NNL-1 | Opioids | ETAZENE |
| Cannabimimetics | 4F-MDMB-BINACA | Opioids | METODESNITAZENE |
| Cannabimimetics | 5F-MN-18 | Opioids | U-49900 |
| Cannabimimetics | 5F-NPB-22 | Opioids | U-51754 |
| Cannabimimetics | 5F-SDB-005 | Opioids | P-HYDROXY-BUTYRYLFENTANYL |
| Cannabimimetics | ADB-BINACA | Opioids | BRORPHIN] |
| Cannabimimetics | AMB | Opioids | U-49900 |
| Cannabimimetics | BIPICANA | Opioids | U-51754 |
| Cannabimimetics | FUB-NPB-22 | Opioids | FLUNITAZENE |
| Cannabimimetics | 4F-MDMB-BICA | PCP-like | 3-CL-PCP |
| Cannabimimetics | 5F-AB-PICA | PCP-like | 3-F-PCP |
| Cannabimimetics | 5F-PY-PICA | PCP-like | 3-MEO-PCE HYDROCHLORIDE |
| Cannabimimetics | AB-BICA | PCP-like | 3-MEO-PCP HYDROCHLORIDE |
| Cannabimimetics | ADB-BICA | PCP-like | DIPHENIDINE |
| Cannabimimetics | NNL-2 | PCP-like | EPHENIDINE |
| Cannabimimetics | JWH-320 | PCP-like | METHOXPHENIDINE |
| Cannabimimetics | EG-2201 | Phenethylamine | 2C-C |
| Cannabimimetics | NNL-1 | Phenethylamine | 2C-E |
| Cannabimimetics | 4F-MDMB-BINACA | Phenethylamine | 5-MAPB |
| Cannabimimetics | 5F-MN-18 | Phenethylamine | 3-MEC |
| Cannabimimetics | 5F-NPB-22 | Phenethylamine | 5-APB |
| Cannabimimetics | 5F-SDB-005 | Phenethylamine | 6-APB |
| Cannabimimetics | ADB-BINACA | Phenethylamines | 5-MBPB[29] |
| Cannabimimetics | AMB | Phenethylamines | ETHYLPHENIDATE |
| Cannabimimetics | BIPICANA | Phenethylamines | 5-MBPB |
| Cannabimimetics | FUB-NPB-22 | PIEDS | NOOPEPT |
| Cannabimimetics | NPB-22 | PIEDS | JNJ-28330835 |
| Cannabimimetics | 5F-AB-PICA | PIEDS | YK-11 |
| Cannabimimetics | 5F-PY-PICA | PIEDS | AMINOTADALAFIL |
| Cannabimimetics | AB-BICA | Prescribed drugs | TIANEPTINE |
| Cannabimimetics | ADB-BICA | Psychostimulants | ETHYLPHENIDATE |
| Cannabimimetics | NNL-2 | Psychostimulants | DIMETHOCAINE |
| Cannabimimetics | JWH-320 | Tryptamines | 5-MEO-EPT |
| Cannabimimetics | MMB-022 | Tryptamines | 5-MEO-MET |
| cathinone | 3-FEA | Tryptamines | 5-MEO-NIPT |
| cathinone | 3-FPM | Tryptamines | 5-MEO-EPT |
| cathinone | 4-CL-PVP | Tryptamines | 5-MEO-MET |
| cathinone | 4-CMC | Tryptamines | 5-MEO-NIPT |
| cathinone | EPHYLONE | Tryptamines | 5-CHLORO-DMT |
| cathinone | ISOPROPYLPHENIDATE | Tryptamines | 5-CHLORO-DMT |
| cathinone | N-ETHYL-HEXEDRONE | Tryptamines | 5-MEO-MET |
| cathinone | NRG-3 | Tryptamines | 5-MEO-NIPT |
| cathinone | 2-NMC | Tryptamines | 4-ACO-MET FUMARATE |
| cathinone | 4-CEC | Tryptamines | 4-HO-MIPT FUMARATE |
| cathinone | 4-MPD | Tryptamines | 5-MEO-DMT |
| cathinone | 4F-PHP | Tryptamines | DPT HYDROCHLORIDE |
| cathinone | DIBUTYLONE | Tryptamines | 4-HO-DPT FUMARATE |
| cathinones | TH-PVP | Tryptamines | EPT FUMARATE SALT |
| Cathinones | N-ETHYL-(NOR)-PENTEDRONE(NEP) | Tryptamines | MET FUMARATE |
| Cathinones | 4-CDC | Tryptamines | MIPT FUMARATE |
| Cathinones | 4H-CMC |  |  |
